# Angioimmunoblastic T-cell lymphoma with extensive follicular dendritic cell and fibroblastic reticular cell network proliferation mimicking follicular dendritic cell sarcoma: A case report with pathologic, immunophenotypic, and molecular findings

**DOI:** 10.3389/fonc.2022.983585

**Published:** 2022-10-24

**Authors:** Fen Zhang, Wenyu Li, Qian Cui, Yu Chen, Yanhui Liu

**Affiliations:** Department of Pathology, Guangdong Provincial People’s Hospital, Guangdong Academy of Medical Sciences, Guangzhou, China

**Keywords:** angioimmunoblastic T-cell lymphoma, follicular dendritic cell, mutation, diagnosis, morphology

## Abstract

Angioimmunoblastic T-cell lymphoma (AITL) is a common type of nodal peripheral T-cell lymphoma, which always presents with extensive follicular dendritic cell (FDC) meshwork. Here, we report a case of AITL combined with extensive spindle cell meshwork. Spindle cells occupied were positive for the FDC markers CD21, CD23, and CD35. Furthermore, some cells were positive for desmin and smooth muscle actin (SMA), suggesting the differentiation of fibroblastic reticular cell (FRC). Interestingly, the proliferation of spindle cells was so extensive that was easily misdiagnosed as FDC sarcoma (FDCS). Next-generation sequencing showed that the common mutations reported in AITL, including *RHOA*, *TET2*, and *IDH2*, were also detected in this case, while the genes that are recurrently mutated in FDCS were not detected. Regrettably, the patient died 19 months later. Overall, we highlight the unusual morphologic features in an AITL patient with extensive FDC and FRC network that may be misdiagnosed as FDCS, and careful morphological observation and immunochemical and molecular examinations are crucial for an accurate diagnosis.

## Introduction

Angioimmunoblastic T-cell lymphoma (AITL) is a rare aggressive malignant tumor derived from mature T-follicular helper (TFH) cells. It is the second most common type of peripheral T-cell lymphomas (PTCLs) and accounts for about 20% (1). Patients with AITL are always diagnosed with advanced stages, and the 5-year survival is only about 30% (2). Generally, the diagnosis of AITL is confirmed by experienced pathologists according to the results of biopsies. The lymph node architecture is partially or totally effaced by small to medium atypical T cells that are typically positive for CD2, CD3, CD4, CD5, CD10, BCL6, C-X-C motif chemokine ligand 13 (CXCL13), ICOS, and programmed cell death 1 (PD-1). The neoplastic cells always congregate near high endothelial venules and are surrounded by expended follicular dendritic cell (FDC) meshwork and inflammatory cell infiltrates (3). However, a small subset of AITL cases still remains difficult to diagnose, and exploitation of the potent supplementary diagnostic methods is of importance.

Nodal FDC sarcoma (FDCS) is a very rare entity usually affecting cervical and abdominal lymph nodes and is generally considered as an indolent tumor with weak aggressiveness (4). Expansion of FDC is common in AITL patients, but discrimination of the unusual expanded FDC meshwork and FDCS is quite difficult. Previously, Benharroch et al. (5) reported a Jewish case who presented with the combination of AITL and FDCS, but the possibility of AITL with an excessively expanded FDC meshwork could not be excluded by the author based on the results of histological examination. Rogges et al. (6) recently reported an Italian patient diagnosed with AITL and FDC expansion, who excluded FDCS based on the results of histopathology and mutation profile. Similarly, we report a Chinese female patient who was diagnosed with AITL with extensive FDC networks mimicking FDCS. She progressed to recurrence and refractory disease and died from AITL after 19 months of the diagnosis.

## Case presentation

**Figure 1:**
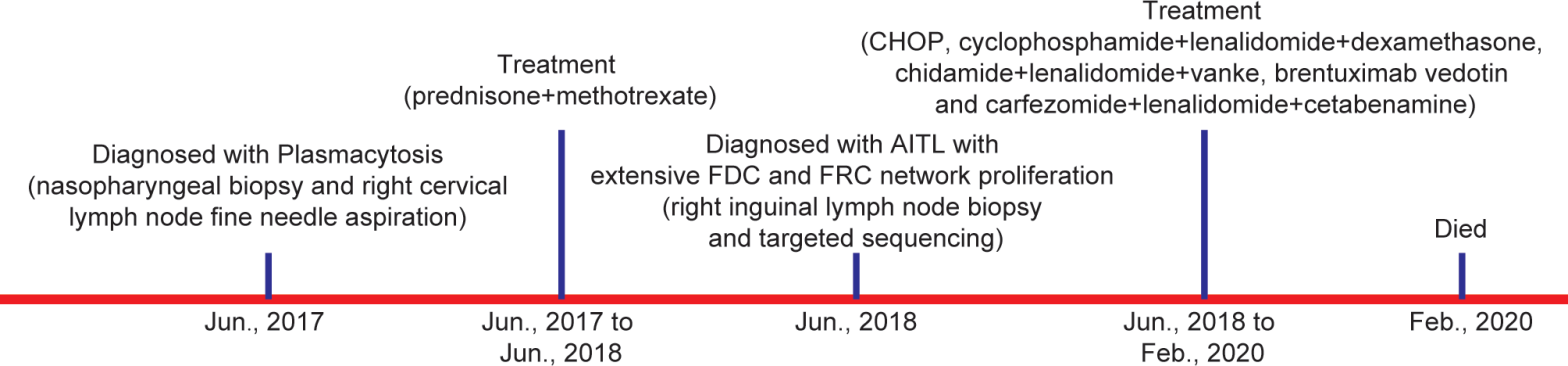
Diagnosis and treatment process of the patient. (AITL, angioimmunoblastic T-cell lymphoma; FDC, follicular dendritic cell; FRC, fibroblastic reticular cell).

A 40–45-year-old woman arrived for consultation with facial swelling that affected eating and breathing as well as the palpable “nodules” in both groins in June 2017. **Figure 1** summarizes the important events of this patient according to the timeline. Generalized lymphadenopathy and multiple nodules in the bilateral parotid glands [standard uptake value (SUV)max 17.2] were found, as examined by the PET-CT. However, nasopharyngeal biopsy and right cervical lymph node fine-needle aspiration showed no evidence of tumor. In addition, no monoclonal bands were found as detected by serum immunofixation electrophoresis. Thus, she was clinically diagnosed as having plasmacytosis and given prednisone and methotrexate. Meanwhile, she received Chinese herbal medicine from another hospital. Cervical lymph node enlargement regressed, while the bilateral inguinal lymph node size remained unchanged after the treatment.

**Figure 2:**
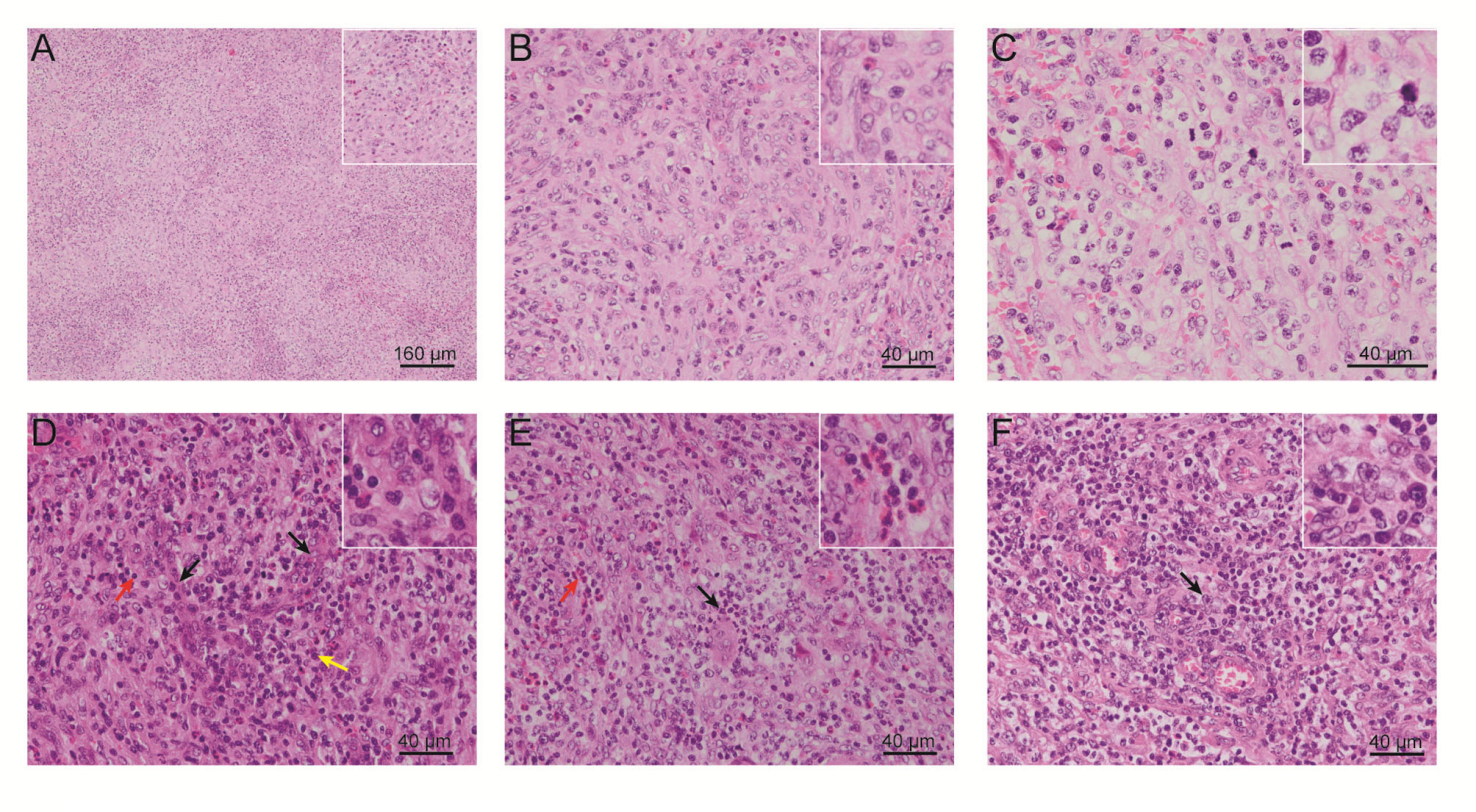
Representative H&E-stained images of the patient’s right inguinal lymph node. **(A)** Cell hyperplasia and perinodal infiltration. **(B)** Vortex distribution of spindle cells. **(C)** Medium-sized lymphoid tumor cells with mild atypia, unclear cell boundary, and abundant pale cytoplasm. Abundant branching of high endothelial small vessels, mixed with **(D)** small lymphocytes and plasma cells, **(E)** eosinophils and neutrophils, and **(F)** few scattered large immunoblastic cells. (H&E, Eosin & Hematoxylin).

**Figure 3:**
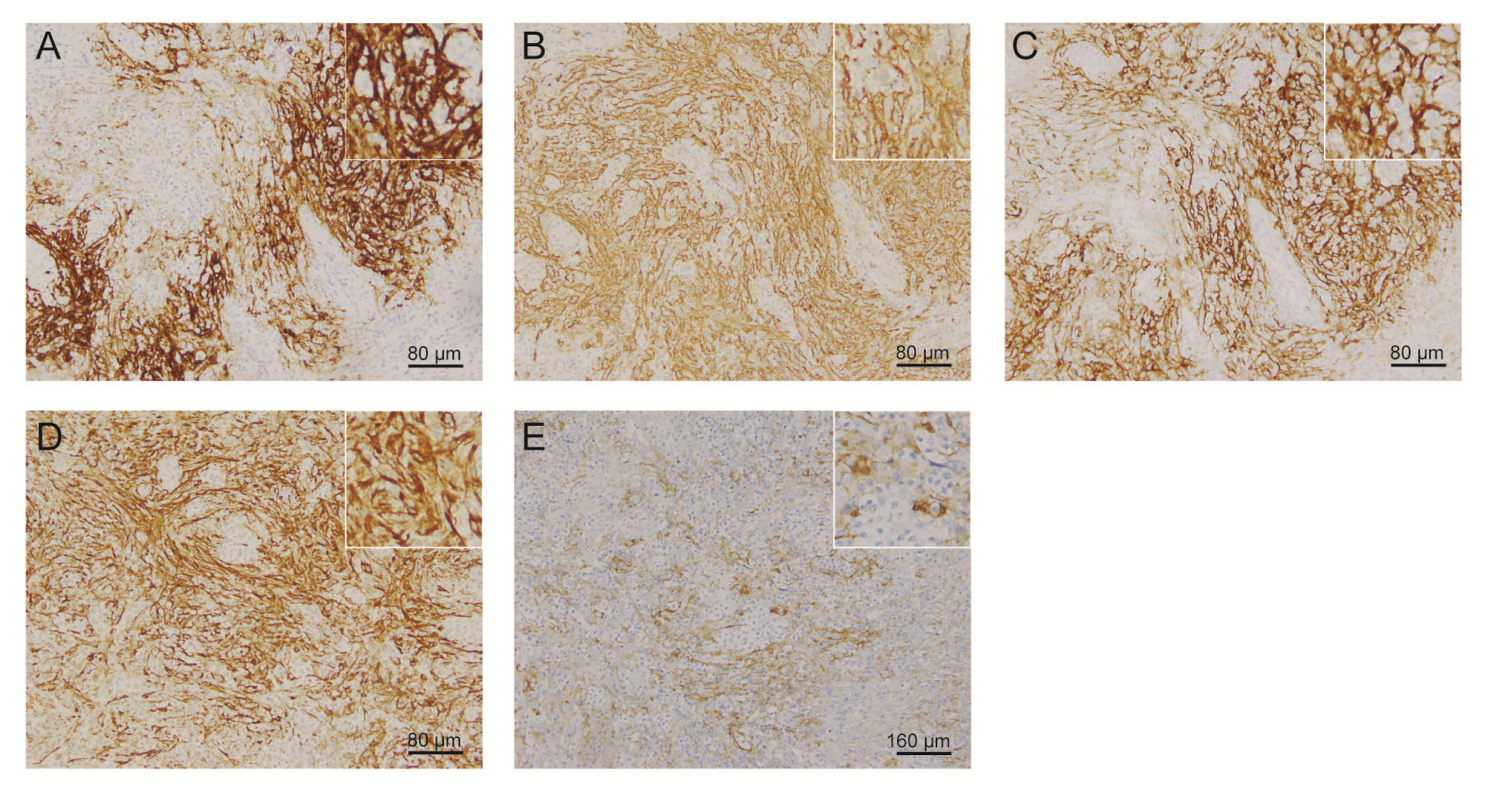
Representative IHC staining images of spindle cells. Spindle cells were positive for **(A)** CD21, **(B)** CD23, **(C)** CD35, **(D)** desmin, and **(E)** SMA. (IHC, immunohistochemistry; SMA, smooth muscle actin).

**Figure 4:**
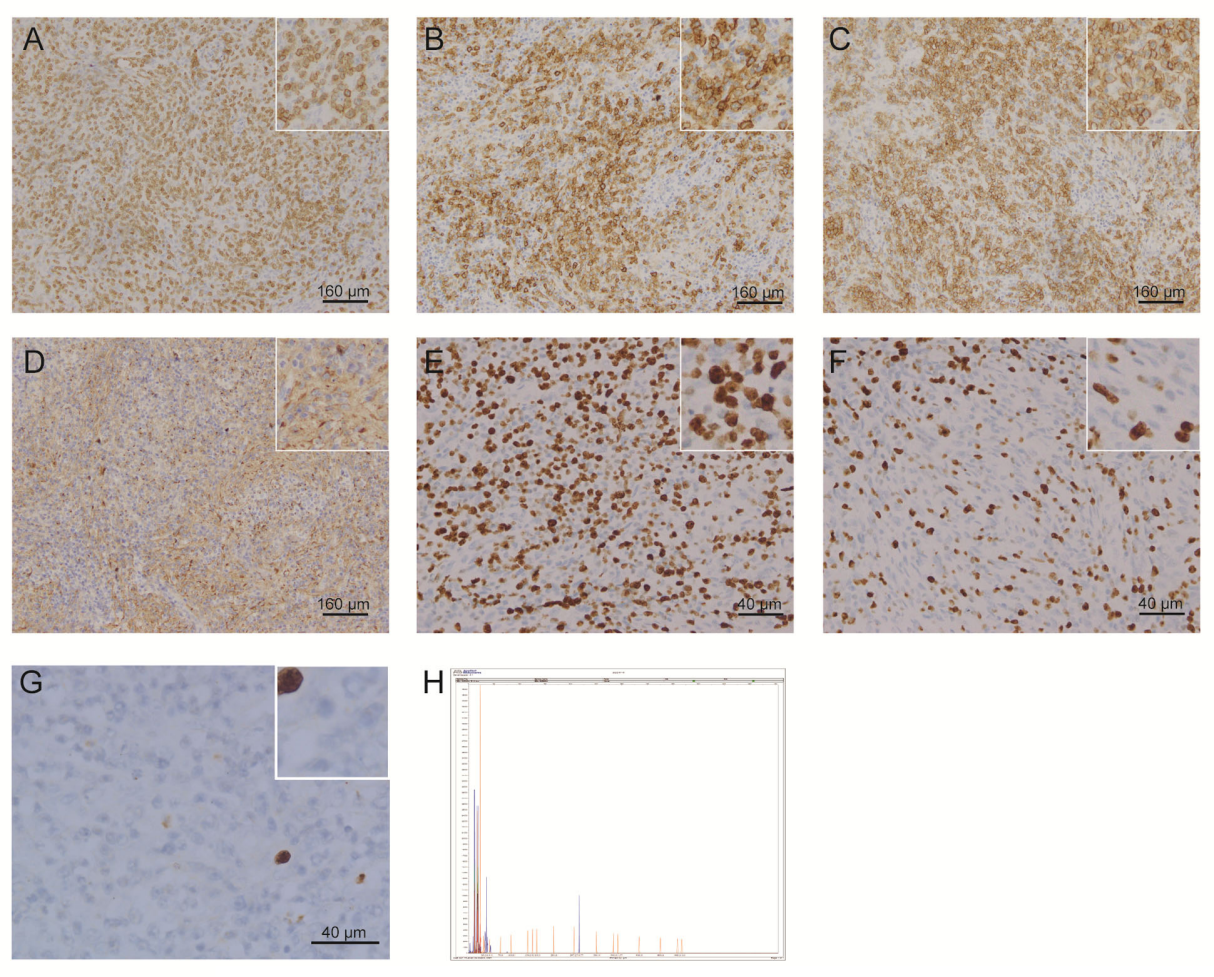
Representative IHC staining images and detection of clonal rearrangement of the *TCR* gene in lymphoid tumor cells. Lymphoid tumor cells were positive for **(A)** CD3, **(B)** CD10, **(C)** PD-1, and **(D)** CXCL13. **(E, F)** Lymphoid tumor cells were positive for Ki-67, while most of the spindle cells were negative for Ki-67 staining. **(G)** A few scattered immunoblastic large cells were positive for EBER. **(H)** Clonal rearrangement of *TCRβ* was observed. (IHC, immunohistochemistry; TCR, T cell receptor; PD-1, programmed cell death 1; CXCL13, C-X-C motif chemokine ligand 13; EBER, Epstein-Barr virus encoded small RNA).

One year later, the patient presented with significantly enlarged inguinal lymph nodes on both sides, increased local skin temperature, local tenderness, surface redness, and left lower extremity edema. A right inguinal lymph node biopsy was carried out. The normal structure of the lymph node was effaced (**Figure 2A**), with hyperplasia of several kinds of cells and perinodal infiltration. In most of the areas, the proliferation of spindle cells was dominant, with vortex distribution (**Figure 2B**). Some areas were dominated by medium-sized lymphoid tumor cells with mild atypia, unclear cell boundary, and abundant pale cytoplasm (**Figure 2C**). Affluent branching of high endothelial small vessels, mixed with small lymphocytes, plasma cells (**Figure 2D**), eosinophils, and neutrophils was seen in the background (**Figure 2E**), together with few scattered large immunoblastic cells (**Figure 2F**). The immunohistochemistry (IHC) staining demonstrated that the spindle cells were positive for complement C3d receptor 2 (CD21) (**Figure 3A**), CD23 (**Figure 3B**), CD35 (**Figure 3C**), and desmin (**Figure 3D**) and partially positive for SMA (**Figure 3E**). The lymphoid tumor cells were positive for CD3 (**Figure 4A**), CD4, CD10 (**Figure 4B**), PD-1 (**Figure 4C**), CXCL13 (**Figure 4D**), and T cell receptor, bF1 (*TCRbF1*) and negative for CD20, CD8, and T cell receptor, g (TCRg). The Ki-67 index of the tumor cells was about 60%, while majority of the spindle cells were negative for Ki-67 staining (**Figures 4E, F**). *In situ* hybridization for Epstein-Barr virus (EBV)-encoded small RNA (EBER) showed that a few scattered immunoblastic large cells were positive for EBER (**Figure 4G**). Clonal rearrangement of *TCRβ* gene was detected (**Figure 4H**).

To further describe the characteristics of this case, we also carried out next-generation sequencing (DNA sequencing) to detect the mutation profile of the lymph node sample. Mutations of 571 lymphoma-related genes (**Supplementary Table S1**) were detected on Novaseq (Illumina, San Diego, CA, USA). Variants, including single-nucleotide variations (SNVs) and Indels (insertion and deletion), were screened by Shanghai Rightongene Biotechnology Co., Ltd. (Shanghai, China), based on the following filter conditions: 1) SNVs or Indels with a variant allele frequency (VAF) ≥1% were retained; 2) SNVs or Indels with a mutation allele frequency (MAF) ≥0.001 in databases of the 1000 Genomes (1KG) Project (7), ExAC_ALL, and ExAC_EAS (8) were removed; 3) SNVs or Indels including stopgain, stoploss, frameshift, non-frameshift, and splicing sites were retained; 4) missense variants that are predicted to be deleterious by SIFT and PolyPhen-2 were retained. According to this, mutations in *RHOA*, *TET2*, *IDH2*, *ERG*, *JAK1*, *EGFR*, *MUC4*, *MYH11*, *PKP2*, *ARHGAP29*, *CCND1*, and *SAMD9* genes were detected (**Table 1**).

**Table 1:** Summary of the results for the targeted NGS screening.

Gene	cDNA change	Protein change	VAF	Minor allele frequency^a^			Functional prediction^b^	
				1KG	ExAC_ALL	ExAC_EAS	SIFT	PolyPhen-2
RHOA	c.50G>T	p.Gly17Val	11.97%	0	0	0	Damaging	Damaging
TET2	c.4138C>T	p.His1380Tyr	9.80%	0.0004	0.0001	0	Damaging	Damaging
TET2	c.5604delT	p.His1868fs	11.52%	0	0	0	–	–
IDH2	c.516G>C	p.Arg172Ser	9.24%	0	0	0	Damaging	Damaging
ERG	c.874A>T	p.Lys292*	38.59%	0	0.00004	0.0005	–	–
JAK1	c.2635C>T	p.Arg879Cys	1.08%	0	0.00002	0.0001	Damaging	Damaging
EGFR	c.2033C>T	p.Thr678Met	7.78%	0	0.00003	0	Damaging	Damaging
MUC4	c.11918G>T	p.Ser3973Ile	40.06%	0	0.0001	0	–	Damaging
MYH11	c.3172C>T	p.Arg1058Trp	45.30%	0	0	0	Damaging	Damaging
PKP2	c.1943T>C	p.Phe648Ser	7.21%	0	0	0	Damaging	Damaging
ARHGAP29	c.221C>A	p.Thr74Asn	35.05%	0	0.00005	0.0006	Damaging	Damaging
CCND1	c.409C>A	p.Leu137Met	47.00%	0	0.00002	0.0004	Damaging	Damaging
SAMD9	c.689G>A	p.Arg230His	43.20%	0.0002	0.0002	0	Damaging	Damaging

a Minor allele frequencies were estimated according to the databases of the 1000 Genomes (1KG) Project, ExAC_ALL, and ExAC_EAS.

b Variant pathogenicity was assessed by the SIFT and PolyPhen-2 tools. *Stopgain. cDNA, complementary DNA; IKG, 1,000 genomes project; ExAC_EAS, ExAC_East Asian.

Based on the results of histological examination and mutational characteristics, the diagnosis of AITL (at a stage of IV) with extensive FDC and fibroblastic reticular cell (FRC) network proliferation was confirmed in July 2018.

The anti-AITL therapeutic schedules for this patient were complex (**Figure 1**). First, she received CHOP (cyclophosphamide, Adriamycin, vincristine, and prednisone) combined with 20 mg chidamide for six cycles. After the first cycle of chemotherapy, skin pruritus was relieved, as well as the swelling of the left lower extremity. However, the disease progressed. Then, she was injected with gemcitabine hydrochloride. Two cycles later, the CT examination indicated that she achieved stable disease (SD). Unluckily, the patient developed fever and infection and was treated with moxifloxacin. Also, she was given cyclophosphamide, lenalidomide, and dexamethasone owing to the increased pleural effusion and poor mental state. Following one cycle of the treatment, she achieved SD but had a cough that was so frequent to lie at night. Then, the treatment regimen was changed into chidamide, lenalidomide, and Velcade. Disappointingly, the disease progressed again, and brentuximab vedotin (a monoclonal antibody of CD30) was given. Three cycles later, the symptoms were not improved, and multiple lung lesions in both lungs were detected. Based on this, she was diagnosed as having recurrent refractory AITL. Subsequently, salvage therapy (carfezomide, lenalidomide, and cetabenamine) was recommended. Cough and sputum were improved after the treatment. Regrettably, she died from AITL in February 2020.

## Discussion

In the present study, we reported a Chinese female patient who was diagnosed as having AITL combined with extensive spindle cell network. This patient was diagnosed as having plasmacytosis without detectable tumor cells 1 year before the diagnosis. Several research groups assessed the behavior of plasma cells in AITL, which ranged from reactive plasmacytosis to striking clonal proliferation (9–11). The performance status of AITL patients with exuberant polyclonal plasmacytosis was significantly worse compared to those in AITL patients without exuberant polyclonal plasmacytosis, but patients with plasmacytosis responded well to chemotherapy and immunosuppressants and had a favorable outcome (10). This case was biopsied twice at the initial onset of disease. However, the nasopharyngeal site was often infected and inguinal lymph nodes presented with more necrotic area, which affected the diagnosis. Thus, how to take biopsy to improve the diagnostic success rate still remains a big challenge for clinicians.

It was uncertain whether the extensive proliferation of spindle cells was an accompanying tumor or not at first. The proliferation of spindle cells was more extensive than other AITL cases, which was easily misdiagnosed as FDCS. In this case, the morphology of spindle cells was gentle without atypia and mitosis, and most of the spindle cells were negative for Ki-67 staining while the Ki-67 index of tumor cells was about 60%. Thus, FDCS was excluded for which the Ki-67 index was always less than 30%. Moreover, parts of the spindle cells expressed desmin and SMA, suggesting that FRCs existed. Similarly, the proliferation of both FDC and FRC in AITL has been reported by Jones et al. (12). Recently, Benharroch et al. (5) reported a Jewish case who was diagnosed with the combination of AITL and FDCS only based on the histopathology. However, the authors acknowledged that they cannot provide sufficient evidence to exclude excessive FDC meshwork proliferation from FDCS. Also, Starkey et al. (13) described an elderly male patient with peripheral T-cell lymphoma (PTCL) accompanied by an excessive FDC network mimicking FDCS. Such cases of extensive FDC hyperplasia challenge pathological examination alone.

Next-generation sequencing emerges as a reliable method for the adjuvant diagnosis of lymphoma. It has been demonstrated that *TET2*, *RHOA^G17V^*, *DNMT3A*, and *IDH2* are the most common mutated genes in AITL, with a mutation frequency of 80% (14–18), 50%–70% (15–19), 20%–40% (15–18, 20), and 20%–30% (15–18, 21), respectively. Among them, *TET2*, *DNMT3A*, and *IDH2* are three genes encoding epigenetic enzymes (18). *RHOA* encodes a small GTPase in either active GTP-bound or inactive GDP-bound forms. The mutation of *RHOA^G17V^* induces the phosphorylation of VAV1 (a guanine exchange factor) and then promotes the enhancement of T-cell receptor (TCR) signaling *in vitro* (22). In this case, mutations in *TET2*, *RHOA^G17V^*, and *IDH2* genes were detected, further confirming the diagnosis of AITL.

Recently, Rogges et al. (6) reported an expansion of FDC in a patient with AITL based on the results of both histopathology and mutational profile. Mutations in genes (i.e., *RHOA*, *DNMT3A*, *TET2*, and *IDH2*) associated with AITL were detected, while the mutations in genes recurrent in FDCS, such as the nuclear factor (NF)κB pathway-involving genes (*BIRC3*, *NFKBIA*, *TRAF3*, *SOCS3*, *CYLD*, and *TNFAIP3*) and tumor suppressor genes (*CDKN2A*, *RB1*, and *TP53*) (23), were detected. Consistently, mutations in *RHOA*, *TET2*, *IDH2*, *ERG*, *JAK1*, *EGFR*, *MUC4*, *MYH11*, *PKP2*, *ARHGAP29*, *CCND1*, and *SAMD9* genes were detected in our patient, none of which was involved in FDCS. These results emphasize the importance of next-generation sequencing in the diagnosis of lymphoma, as well as the differential diagnosis of the expanded FDC meshwork and FDCS.

This patient received various anticancer regimens, including CHOP, gemcitabine hydrochloride, chidamide, lenalidomide, Velcade, and monoclonal antibodies against CD30. However, the curative effect was unsatisfactory, the disease progressed, and the patient died, suggesting a poor prognosis of this case with AITL and extensive FDC and FRC networks.

Taken together, this study described a rare case of AITL combined with extensive FDC and FRC networks mimicking FDCS in a female patient. This case highlights the heterogeneity of the clinical presentation of AITL, and a combination of histological examination and next-generation sequencing may help the clinical diagnosis and initiate treatment of this kind of disease. It is crucial to include more of the same cases to summarize the features of AITL combined with extensive FDC and FRC networks and thereafter to develop efficient treatment methods.

## Data availability statement

The original contributions presented in the study are included in the article/**Supplementary Material**. Further inquiries can be directed to the corresponding author.

## Ethics statement

The studies involving human participants were reviewed and approved by Guangdong Provincial People’s Hospital/Guangdong Academy of Medical Sciences. The patients/participants provided their written informed consent to participate in this study. Written informed consent was obtained from the individual(s) for the publication of any potentially identifiable images or data included in this article.

## Author contributions

YL, FZ, and WL contributed to the study conception and design. Material preparation, data collection and analysis were performed by YL, FZ, WL, QC, YC, and YL. The first draft of the manuscript was written by FZ and WL. All authors reviewed the manuscript. All authors contributed to the article and approved the submitted version.

## Funding

This study was supported by Natural Science Foundation of Guangdong Province (no. 2019A1515011643).

## Acknowledgments

We thank Shanghai Rightongene Biotechnology Co. Ltd. (Shanghai, China) for assisting in molecular detection.

## Conflict of interest

The authors declare that the research was conducted in the absence of any commercial or financial relationships that could be construed as a potential conflict of interest.

## Publisher’s note

All claims expressed in this article are solely those of the authors and do not necessarily represent those of their affiliated organizations, or those of the publisher, the editors and the reviewers. Any product that may be evaluated in this article, or claim that may be made by its manufacturer, is not guaranteed or endorsed by the publisher.
