# Supplementary material for: Angioimmunoblastic T-cell lymphoma with extensive follicular dendritic cell and fibroblastic reticular cell network proliferation mimicking follicular dendritic cell sarcoma: A case report with pathologic, immunophenotypic, and molecular findings
Source: Front Oncol. 2022 Oct 24;12:983585. doi: 10.3389/fonc.2022.983585 (PMC9638041; doi:10.3389/fonc.2022.983585)
Supplement: Supplementary file 1 [file Table_1.pdf]

**Supplemantary Table 1** Lymphoma-related 571 gene list				
ABL1	*EPCAM*	*KMT2C*	*PTP4A1*	*VHL*
ACVR1	*EPHA3*	*KMT2D*	*PTPN11*	*VTCN1*
AGO2	*EPHA5*	*KNSTRN*	*PTPRD*	*WHSC1*
AKT1	*EPHA7*	*KRAS*	*PTPRS*	*WHSC1L1*
AKT2	*EPHB1*	*LATS1*	*PTPRT*	*WT1*
AKT3	*ERBB2*	*LATS2*	*RAB35*	*WWTR1*
ALK	*ERBB3*	*LMO1*	*RAC1*	*XIAP*
ALOX12B	*ERBB4*	*LYN*	*RAC2*	*XPO1*
AMER1	*ERCC2*	*MALT1*	*RAD21*	*XRCC2*
ANKRD11	*ERCC3*	*MAP2K1*	*RAD50*	*YAP1*
APC	*ERCC4*	*MAP2K2*	*RAD51*	*YES1*
AR	*ERCC5*	*MAP2K4*	*RAD51B*	*ZFHX3*
ARAF	*ERF*	*MAP3K1*	*RAD51C*	*ACVR1B*
ARID1A	*ERG*	*MAP3K13*	*RAD51D*	*ARFRP1*
ARID1B	*ERRFI1*	*MAP3K14*	*RAD52*	*BCL2L2*
ARID2	*ESR1*	*MAPK1*	*RAD54L*	*BCORL1*
ARID5B	*ETV1*	*MAPK3*	*RAF1*	*BTG1*
ASXL1	*ETV6*	*MAPKAP1*	*RARA*	*BTG2*
ASXL2	*EZH1*	*MAX*	*RASA1*	*C11orf30*
ATM	*EZH2*	*MCL1*	*RB1*	*CD22*
ATR	*FAM175A*	*MDC1*	*RBM10*	*CD70*
ATRX	*FAM46C*	*MDM2*	*RECQL*	*CDKN2A*
AURKA	*FAM58A*	*MDM4*	*RECQL4*	*CTNNA1*
AURKB	*FANCA*	*MED12*	*REL*	*CUL4A*
AXIN1	*FANCC*	*MEF2B*	*RET*	*CYP17A1*
AXIN2	*FAT1*	*MEN1*	*RFWD2*	*DDR1*
AXL	*FBXW7*	*MET*	*RHEB*	*EPHB4*
B2M	*FGF19*	*MGA*	*RHOA*	*FANCG*
BABAM1	*FGF3*	*MITF*	*RICTOR*	*FANCL*
BAP1	*FGF4*	*MLH1*	*RIT1*	*FAS*
BARD1	*FGFR1*	*MPL*	*RNF43*	*FGF10*
BBC3	*FGFR2*	*MRE11A*	*ROS1*	*FGF12*
BCL10	*FGFR3*	*MSH2*	*RPS6KA4*	*FGF14*
BCL2	*FGFR4*	*MSH3*	*RPS6KB2*	*FGF23*
BCL2L1	*FH*	*MSH6*	*RPTOR*	*FGF6*
BCL2L11	*FLCN*	*MSI1*	*RRAGC*	*GABRA6*
BCL6	*FLT1*	*MSI2*	*RRAS*	*GATA4*
BCOR	*FLT3*	*MST1*	*RRAS2*	*GATA6*
BIRC3	*FLT4*	*MST1R*	*RTEL1*	*GID4*
BLM	*FOXA1*	*MTOR*	*RUNX1*	*GNA13*
BMPR1A	*FOXL2*	*MUTYH*	*RXRA*	*GRM3*
BRAF	*FOXO1*	*MYC*	*RYBP*	*HDAC1*
BRCA1	*FOXP1*	*MYCL1*	*SDHA*	*HSD3B1*
BRCA2	*FUBP1*	*MYCN*	*SDHAF2*	*IRF2*
BRD4	*FYN*	*MYD88*	*SDHB*	*KEL*
BRIP1	*GATA1*	*MYOD1*	*SDHC*	*KLHL6*
BTK	*GATA2*	*NBN*	*SDHD*	*LTK*
CALR	*GATA3*	*NCOA3*	*SESN1*	*MAF*
CARD11	*GLI1*	*NCOR1*	*SESN2*	*MERTK*
CARM1	*GNA11*	*NEGR1*	*SESN3*	*MKNK1*
CASP8	*GNAQ*	*NF1*	*SETD2*	*MTAP*
CBFB	*GNAS*	*NF2*	*SETD8*	*MYCL*
CBL	*GPS2*	*NFE2L2*	*SF3B1*	*NT5C2*

CCND1	GREM1	NFKBIA	SH2B3	P2RY8
CCND2	GRIN2A	NKX2-1	SH2D1A	PARP2
CCND3	GSK3B	NKX3-1	SHOC2	PARP3
CCNE1	H3F3A	NOTCH1	SHQ1	PDK1
CD274	H3F3B	NOTCH2	SLX4	PIK3C2B
CD276	H3F3C	NOTCH3	SMAD2	PPP2R2A
CD79A	HGF	NOTCH4	SMAD3	PTPRO
CD79B	HIST1H1C	NPM1	SMAD4	QKI
CDC42	HIST1H2BD	NRAS	SMARCA4	SGK1
CDC73	HIST1H3A	NSD1	SMARCB1	SNCAIP
CDH1	HIST1H3B	NTHL1	SMARCD1	TIPARP
CDK12	HIST1H3C	NTRK1	SMO	TYRO3
CDK4	HIST1H3D	NTRK2	SMYD3	ZNF217
CDK6	HIST1H3E	NTRK3	SOCS1	ZNF703
CDK8	HIST1H3F	NUF2	SOS1	ACTB
CDKN1A	HIST1H3G	NUP93	SOX17	BCLAF1
CDKN1B	HIST1H3H	PAK1	SOX2	CD36
CDKN2Ap14A	HIST1H3I	PAK7	SOX9	CD58
CDKN2Ap16I	HIST1H3J	PALB2	SPEN	CHD2
CDKN2B	HIST2H3C	PARK2	SPOP	CIITA
CDKN2C	HIST2H3D	PARP1	SPRED1	DDX3X
CEBPA	HIST3H3	PAX5	SRC	DTX1
CENPA	HLA-A	PBRM1	SRSF2	DUSP2
CHEK1	HLA-B	PDCD1	STAG2	EBF1
CHEK2	HNF1A	PDCD1LG2	STAT3	EGR2
CIC	HOXB13	PDGFRA	STAT5A	HIST1H1E
CREBBP	HRAS	PDGFRB	STAT5B	HIST2H2AB
CRKL	ICOSLG	PDPK1	STK11	IGLL5
CRLF2	ID3	PGR	STK19	IKBKB
CSDE1	IDH1	PHOX2B	STK40	IRF8
CSF1R	IDH2	PIK3C2G	SUFU	ITPKB
CSF3R	IFNGR1	PIK3C3	SUZ12	KIR2DL1
CTCF	IGF1	PIK3CA	SYK	KRT20
CTLA4	IGF1R	PIK3CB	TAP1	LRRN3
CTNNB1	IGF2	PIK3CD	TAP2	MAPK7
CUL3	IKBKE	PIK3CG	TBX3	MPEG1
CXCR4	IKZF1	PIK3R1	TCEB1	MYOM2
CYLD	IL10	PIK3R2	TCF3	NCOR2
CYSLTR2	IL7R	PIK3R3	TCF7L2	NFKBIE
DAXX	INHA	PIM1	TEK	NRXN3
DCUN1D1	INHBA	PLCG2	TERT	NSD2
DDR2	INPP4A	PLK2	TET1	PCLO
DICER1	INPP4B	PMAIP1	TET2	POSTN
DIS3	INPPL1	PMS1	TGFBR1	POT1
DNAJB1	INSR	PMS2	TGFBR2	PTPN6
DNMT1	IRF4	PNRC1	TMEM127	RAG1
DNMT3A	IRS1	POLD1	TMPRSS2	RAG2
DNMT3B	IRS2	POLE	TNFAIP3	RIPK1
DOT1L	JAK1	PPARG	TNFRSF14	ROBO2
DROSHA	JAK2	PPM1D	TOP1	SAMHD1
DUSP4	JAK3	PPP2R1A	TP53	SIN3A
E2F3	JUN	PPP4R2	TP53BP1	SLITRK3
EED	KDM5A	PPP6C	TP63	STAT6
EGFL7	KDM5C	PRDM1	TRAF2	TBL1XR1

EGFR	*KDM6A*	*PRDM14*	*TRAF7*	*TGM7*
EIF1AX	*KDR*	*PREX2*	*TSC1*	*TLDC2*
EIF4A2	*KEAP1*	*PRKAR1A*	*TSC2*	*TMSB4X*
EIF4E	*KIT*	*PRKCI*	*TSHR*	*TYK2*
ELF3	*KLF4*	*PRKD1*	*U2AF1*	*UBE2A*
EP300	*KMT2A*	*PTCH1*	*UPF1*	*ZFP36L1*
EPAS1	*KMT2B*	*PTEN*	*VEGFA*	*ZMYM3*
ZNF608				
